# Practice of walking and its association with perceived environment among elderly Brazilians living in a region of low socioeconomic level

**DOI:** 10.1186/1479-5868-7-67

**Published:** 2010-09-17

**Authors:** Emanuel P Salvador, Rodrigo S Reis, Alex A Florindo

**Affiliations:** 1 School of Public Health/Department of Nutrition/University of Sao Paulo, Brazil; 2 School of Physical Education/Pontiff Catholic University of Parana, Brazil; 3 Federal University of Parana, Brazil; 4 School of Arts, Sciences and Humanities/University of Sao Paulo, Brazil

## Abstract

**Background:**

The aim of this study was analyze associations between the practice of walking and environmental perception among elderly Brazilians in a region of low socioeconomic level.

**Methods:**

A cross-sectional study was conducted among 385 elderly people aged 60 years and over. To evaluate walking, the International Physical Activity Questionnaire (IPAQ), long version (leisure and transport modules) was used. The environment was evaluated by means of the Neighborhood Environmental Walkability Scale (NEWS) (adapted Brazilian version). For the statistical analysis, multiple logistic regression models were created separately for men and women. The practice of at least 150 minutes a week of walking was the dependent variable, and the variables of environmental perception were the independent variables. All the models were controlled for schooling level and age.

**Results:**

The proportion of elderly people active in walking was 56.9% for the men and 26.4% for the women. The perception of the presence of soccer fields (OR = 4.12) and their proximity, within ten minutes' walk from home (OR = 3.43), were associated with the practice of walking among the men. The perception of the presence of public squares (OR = 4.70) and the proximity of primary healthcare units, within ten minutes' walk from home (OR = 3.71), were associated with the practice of walking among the women. An association with adequate perception of vehicle traffic remained at the threshold of significance for the women.

**Conclusion:**

Accessibility of leisure structures such as football fields and public squares and of health services such as primary healthcare units were important environmental variables associated with the practice of walking among elderly people living in a region of low socioeconomic level in Brazil. These variables need to be taken into consideration when aiming to promote the practice of walking among elderly people living in similar regions.

## Background

Although the participation in physical activities is related to many benefits for health, studies have indicated high prevalence of inactive elderly people in different countries around the world [1].

Walking is one of the most accessible forms of physical activity that can be incorporated into individuals' day-to-day routines, especially for the elderly population. Studies have shown that this type of physical activity is the one most practiced by elderly people [2,3]. However, a variety of factors such as age, sex, schooling level, smoking habit and income level influence such practices [4-6]. In addition to these variables, some recent studies have shown that the environment also influences the engagement in walking among the elderly [7-9].

The ecological model proposed by Sallis et al.[10] showed that modification on behavioral patterns to increase physical activity is difficult and does not depend solely on individuals but also on the environment within which they live. The environment includes different levels and variety of variables affecting an individual's behavior such as demographic, biological, psychological and family situation (first level), perceptions of safety, attractiveness, comfort, crime, facilities and conveniences (second level), the structure of the neighborhood, transport system and various services facilities that may influence physical behavior such as workplaces, schools, climate, topography, open public spaces, air quality, social networks, social capital, primary healthcare units and equipment and installations for leisure and recreation (third level) and the local, state and national public policies (fourth level).

It is known that esthetics, accessibility, perceptions of general safety and safety relating to traffic, and the presence of parks, public squares, gyms and primary healthcare units are associated with the engagement in walk [9]. King [11] showed that perceived factors such as crime safety and social cohesion were associated with physical activity in elderly living in Denver, United States. Nevertheless, there are still no studies on which aspects of the environment might be associated with the participation in walk, among elderly people living in regions of low socioeconomic level in middle-income countries such as Brazil. A recent review demonstrated that the less affluent population groups as most exposed to environmental risks in the place of residence (e.g. dampness, traffic related pollution and noise) [12]. Because these environmental aspects are recognized as potential barriers for walking [9] it is important to explore their association in the present context.

Therefore, the objective of this study was to investigate which variables relating to the perceived environment might be associated with the participation in walk, among elderly Brazilians living in a region of low socioeconomic level.

## Methods

This was a cross-sectional population-based study conducted among a representative sample of elderly people living in the Ermelino Matarazzo district. This is located in the eastern zone of the municipality of São Paulo, which is the most heavily populated region of the city, with the greatest inequalities related to the socioeconomic level in the city. Ermelino Matarazzo has around 115,571 inhabitants, in an area of 8.9 km^2^, and thus a population density of 12,913. inhabitants per km^2^. According to data from the Foundation for the State Data Analysis System (*Fundação Sistema Estadual de Análise de Dados*, SEADE), only 9.5% of the population living in Ermelino Matarazzo had an income greater than 10 minimum monthly salaries, compared with 21.1% for the whole of the municipality of São Paulo in 2007(Brazilian minimum salary ≅ $270.00).

The last demographic census, which was carried out by the Brazilian Institute for Geography and Statistics (*Instituto Brasileiro de Geografia e Estatística*, IBGE) in 2000, showed that Ermelino Matarazzo was composed of 143 census tracts. Based on this information, the sampling process was performed as a three-stage process. In the first stage was done with probability proportional to size measures (were drawn 35 census tracts out of the 143 possible). In the second-stage were drawn 53 households for census tracts. And in the third-stage were drawn the subjects. The draw the subjects within each household, the Kish methodology was used [13]: this defines random tables according to the number of people in the household. From this, each household received a table defining which subject should be interviewed, according to the number of elderly residents and decreasing order of age among them and only one elder was selected in each household.

The elderly people needed to be 60 years of age or over and to have lived for at least six months at the address that was drawn. Elderly people who presented problems that might have affected their physical activities during the week preceding the interview (e.g. fractures and stroke), or who were unable to answer the questionnaire on their own, were excluded.

For the purpose of calculating the sample size we use the following equation for proportions estimation[14].

$$n_{0}=\frac{P\cdot (1-P)}{{\left(d/z\right)}^{2}}\cdot deff$$

Where:

• P was the proportion of individuals to be estimated for engagement in physical activities. Based on data from a health survey [15] carried out in the municipality of São Paulo, the value of 0.15 was used for this parameter, because in that study, the prevalence of individuals who did not attain the recommendations of at least 150 minutes of physical activity as a means of leisure activity or transport was found to be 85%;

• Among the other parameters: z = 1.96, which was the value on the reduced normal curve corresponding to the confidence level of 95% that was used to determine the confidence interval;

• d = 0.065, which was the sampling error accepted;

• deff = 2.6, which was the design effect based on a previous health survey conducted in the region [15];

By applying these values in the formula, the sample size was calculated to be a minimum of 300 elderly individuals.

A total of 2,309 households were visited, of which 1,985 were successfully contacted. Among these households (1,985) only 530 had elderly people living in and the response rate was 72.6%. After this process 385 elderly people were included and interviewed. Further details related to sample design can be obtained in Salvador et al.[16].

### Measurement of walking

Walking was measured using the long version of the International Physical Activity Questionnaire (IPAQ) which was used as part of a health survey conducted in the municipality of São Paulo in 2003 [15]. Evidence showing that the IPAQ is valid and reproducible among elderly people in Brazil had been produced by Benedetti et al[17], who found reproducibility values of r = 0.95 for repeated measurements with an interval of 21 days, and a Spearman correlation coefficient of r = 0.38 from comparisons using a physical activity diary and r = 0.24 from comparisons using pedometers.

To calculate the minutes walked, the modules of transport-related walking and leisure-time walking were summed. Elderly people who practiced at least 150 minutes of walking per week were considered active. Elderly people that practiced between 10 to 149 minutes of walking per week were considered insufficiently active and elderly people that practiced less 10 minutes of walking per week were considered inactive [18].

### Measurement of perceived environmental characteristics

To evaluate the perceived environment, an adapted version of the Neighborhood Environmental Walkability Scale (NEWS) [19] was used. This had previously been validated by Malavasi et al. [20] (Brazilian version). The scale underwent certain modifications to make it easier to comprehend by elderly people living in Ermelino Matarazzo.

The final adapted version was discussed with specialists in the field of the environment and physical activity in Brazil, and was composed of 38 questions.

The first part of the questionnaire was structured such that the subjects would state how long it would take them to walk from their homes to different commercial, service or leisure establishments in the district where they lived (parks, public squares, places to walk, gyms, clubs, sports courts, soccer fields, bus stops, train stations, health clinics, pharmacies, churches or religious temples, bakeries, bank branches, bars, street fairs, stores, markets and supermarkets). The second part of the questionnaire consisted of items describing the surroundings of the participant's homes. These included presence and quality of pavements/sidewalks and green areas; streets steepness; presence of litter; open-air sewers; heavy traffic; whether there were pedestrian crossings close to their homes; whether drivers usually respected pedestrians on crossings; whether there was any smoke pollution close to their homes; whether the streets close to their homes were well lit at night; whether it was considered safe to walk during the day and night in the vicinity of their homes; whether they received invitations from friends, neighbors and relatives to walk, cycle or do sports in the district, whether guided sports events and/or walks took place in the district; whether the weather (cold, rain or heat) made it difficult to walk, cycle or do sports in the district; and whether the interviewees had dogs and, if so, whether they went out walking with the dog. The subjects were instructed to consider that places were close if they could reach them by walking for not more than ten minutes.

Analysis of the test-retest reliability with one week interval in a sample of 31 elderly, indicated good results for scores that were elaborated with base in individual questions (correlation coefficients ≥ 0.70).

This study was approved by the Research Ethics Committee of the School of Public Health, University of São Paulo. All the elderly people who underwent the physical evaluation received in return explanatory materials about preventive examinations and guidance about Alzheimer's disease, quality of sleep, practice of physical activities and healthy eating, and a booklet about elderly people's rights.

### Statistical analysis

All the study variables were analyzed descriptively by means of absolute and relative frequencies, stratified by sex. We gender stratified all the analysis because the outcome variable (physical activity) was different between men and women and therefore some level of interaction is expected. The chi-square test was used to investigate difference in walking levels between men and women.

Bivariate regression analyses were performed between the practice of at least 150 minutes a week of walk (dependent variable) and the environmental variables (independent variables). The variables that presented p-values < 0.20 were selected for constructing the multiple model [21], and these were adjusted for age and education level. Only the variables for which significance levels of p < 0.05 were obtained after the adjustment were considered significantly associated with the practice of walking among the elderly people.

All the analyses were performed taking into consideration the complex sampling, using primary sampling units and weightings, and were carried out using the Complex Samples module of the Statistical Package for the Social Sciences (SPSS) software, version 15.0.

## Results

Out of the 385 elderly people interviewed, 60.5% were women, 57.1% were aged between 60 and 74 years, 55.5% had white skin color, 54.2% did not have a partner, 78.4% were not working, 86.6% were nonsmokers and 47.6% had attended school for up to four years. The sociodemographic variables of this sample of elderly people from Ermelino Matarazzo were very similar to a representative sample of elderly people from the whole of the municipality of São Paulo (Table 1) [22].

**Table 1:** Socio-demographic data of the District of Ermelino Matarazzo (2007) and the municipality of São Paulo (2003)

Socio-demographic data	Ermelino Matarazzo	São Paulo*
Gender (women)	60.5%	58.6%
Age (60-74 years)	57.1%	77.9%
Race (white)	55.5%	67.5%
Status (single)	54.2%	43.1%
Ocupation (retired)	78.4%	71.4%
Smoke (nonsomkers)	86.6%	84.1%
Education (less than 4 years)	47.6%	46.4%

Source: Project SABE (Health, Welfare and Aging in the city of São Paulo)

With regard to the participation in walk, most of the men were classified as active. There were significant differences between the sexes at the three classification levels with more men classified as active than women (Table 2).

**Table 2:** Classification of levels of walking-related physical activity by gender in elderly of the Municipality of São Paulo, Southeastern, Brazil, 2007

Walking	Gender				TOTAL	p
	Men		Women			
	**n**	**% (95%CI)**	**n**	**% (95%CI)**		
	
Inactive*	13	9.1 (5.1-15.6)	38	16.1 (10.8-23.3)	51	0.080
Insufficiently active*	53	34.0 (26.3-42.6)	118	49.8 (40.5-59.1)	171	0.004
Active*	86	56.9 (47.6-65.8)	77	34.1 (26.4-42.7)	163	< 0.001
TOTAL	152	100.0%	233	100.0%	**385**	

*active: least 150 minutes of walking per week; insufficiently active among 10 until 149 minutes of walking per week; inactive: less 10 minutes of walking per week.

Among the 38 environmental variables evaluated, only seven presented p values < 0.20 for the men and after including the adjustment variables, perceptions of the presence of soccer fields in the district and their proximity to the subjects' homes were found to be significantly associated with the recommended practice of walking, among men (Table 3).

**Table 3:** Final multiple logistic regression model of recommended levels of walking in elderly men. Municipality of São Paulo, Southeastern Brazil, 2007.(n = 152)

Perceived Environmental Variables *	OR	95%CI		p
		Lower	Upper	
Good perception of safety during the night	1.53	0.41	5.61	0.514
Presence of soccer fields in the district	4.12	1.41	12.02	0.011
Walking time of not more than 10 minutes from home to a soccer field	3.43	1.46	8.10	0.006
Absence of open-air sewers	2.18	0.56	8.52	0.253
Presence of places for walking in the district	2.23	0.67	7.40	0.181
Having a pet dog	2.60	0.89	7.63	0.080
Presence of public lighting at night	2.00	0.45	8.90	0.353

* adjusted for age and education. OR = Odds Ratio

For women, nine environmental variables presented p < 0.20 and after including the adjustment variables, the perceptions of public squares in the district and primary healthcare units within 10 minutes' walk from the subjects' homes were found to be significantly associated with the recommended practice of walking, among the women. On the other hand, the perception that the traffic did not get in the way of practicing physical activities remained at the threshold of significance (p = 0.052) (Table 4).

**Table 4:** Final multiple logistic regression model of recommended levels of walking in elderly women. Municipality of São Paulo, Southeastern Brazil, 2007.(n = 233)

Perceived Environmental Variables *	OR	95%CI		p
		Lower	Upper	
Walking time of not more than 10 minutes from home to a bar	4.05	0.27	60.53	0.299
Presence of square	4.70	1.43	15.43	0.012
Perception that drivers respected pedestrian crossings on streets	5.29	0.90	31.10	0.064
Absence of smoke pollution	1.77	0.75	4.17	0.186
Walking time of not more than 10 minutes from home to a soccer field	1.58	0.64	3.90	0.307
Walking time of not more than 10 minutes from home to a pharmacies	2.45	0.88	6.81	0.084
Presence of bar	2.28	0.13	41.25	0.566
Walking time of not more than 10 minutes from home to a primary healthcare	3.71	1.19	11.54	0.025
The traffic was not a barrier against practicing physical activities	2.88	0.99	8.41	0.052

* adjusted for age and education. OR = Odds Ratio

## Discussion

Accessibility of leisure areas such as soccer fields and public squares and accessibility of primary healthcare units were the variables associated with the recommended practice of walking among these elderly Brazilians living in a region of low socioeconomic level.

In our review of the literature, we did not find any studies on the relationship between the environment and the engagement in walk among elderly people living in regions similar to Ermelino Matarazzo.

The proportion of physically active subjects in this sample differed from the findings in some studies already published in developed countries. In a recent study published by Shibata et al. [6], which was conducted among 5,117 adults and elderly people in Japan, the observed prevalence of active women was 28.0% and the prevalence of active men was 31.4%. Panter and Jones [8] found that 18.8% of a population of elderly people in Norwich, England, attained the recommendations for practicing physical activities, by means of walking. Comparing the present study with a survey conducted in Brazil with a sample of adults and elderly people in the municipality of Pelotas [23], the prevalence of elderly people who were active through walking was slightly lower in the Pelotas sample (40.7% among elderly people up to 69 years of age and 31.4% among elderly people aged 70 years and over). The main explanation for these differences lies in the low socioeconomic level of the district of Ermelino Matarazzo, where people walk more as a means of transport than in other localities in Brazil itself and in high-income countries such as Japan and England.

The perceptions of both the presence and the proximity of soccer fields to the elderly men's homes presented significant associations with the practice of walking. In countries like Brazil, soccer fields are leisure spaces that are commonly present, both for practicing this sport and for entertainment. According to national estimates [24,25], more than 30 million people play soccer in Brazil and more than 100 million people are spectators, and the vast majority of them are men. These data may explain the significant association between the practice of walking and the presence of leisure localities relating to playing soccer in districts like Ermelino Matarazzo.

Public squares are public structures that are organized for leisure, with green areas and equipment. Many such areas in peripheral districts like Ermelino Matarazzo in the city of São Paulo have a setup favoring the practice of walking. Some other studies conducted with samples of adults and elderly people have found similar associations between perception of green areas or parks with the practice of walking. Boehmer et al. [26] studied 2,210 adults and elderly people in 13 communities in Arkansas, Missouri and Tennessee, in the United States, and found a significant association between perception of the presence of parks and the practice of walking during leisure time (OR = 2.21; 95% CI 1.50-3.28). Huston et al. [27] investigated 1,796 adults and elderly people in seven states in the United States and found a similar association between perception of the presence of parks and practices of physical activities (OR = 1.51; 95% CI 1.00-2.28).

Objective assessments of the environment have corroborated these results. Cohen et al. [28] studied 1,318 adults and elderly people in the United States and found an association between the presence of parks less than one mile away from the subjects' homes and practices of physical activities during leisure time. Therefore, the results relating to the association between accessibility/proximity of structures with green areas such as public squares and the practice of walking, among the elderly people in Ermelino Matarazzo, were similar to the findings from samples in high-income countries like the United States. This shows that structures of this type are also important in regions of low socioeconomic level.

Primary healthcare units were important because they were significantly associated with the practice of walking among the women. In our review of the literature, we did not find any similar study. This result can be explained by the strategies for promoting physical activities that are implemented in different primary healthcare units through the Brazilian National Health System, in many peripheral districts like Ermelino Matarazzo, where it is very common to organize group walks.

Although vehicle traffic was not considered to be a barrier against practicing physical activities, it was at the threshold of significance with regard to the association with the practice of walking. São Paulo is a city with a fleet of more than 6.5 million vehicles, and this creates barriers against the practice of walking, since excessive numbers of vehicles are correlated with accidents, including knocking down pedestrians, along with huge production of pollutants. There is still a great degree of disrespect for pedestrians among motorists in countries like Brazil. A study conducted in Australia among 1,803 adults and elderly people showed that individuals who had an adequate perception of vehicle traffic had a greater chance of practicing walking (OR = 1.16; 95% CI 1.01-1.56) [28].

Certain limitations of the present study need to be highlighted. The limitation is one that is common to all cross-sectional studies, in which cause-effect relationships cannot be established and the possibility of reverse causality cannot be ruled out. The second limitation is referent to the sample size. The sample was calculated to estimate the prevalence of physical activity and not for testing the association between environment and physical activity. For this reason those results in the limit of the significance were more likely to be affected by this limitation. The third limitation lies in the fact that the information on the environment was gathered through perceptions. Thus, it is possible that these measurements may not represent the real availability or attributes and structures in the district of Ermelino Matarazzo. In accordance to this assumption the literature demonstrates that some discrepancy between observed and perceived environmental measures are expected, particularly distance to destination [29]. However, more recent evidence showed that objective and perceived walkability attributes are well correlated [30]. Similar results are supported by others [31,32]. Perceived evaluations are of great importance because they represent how the people living in a community see the environment within which they live. Along with this characteristic perceptions about the environment are also affected by psychosocial correlates of physical activity (e.g. self-efficacy) [33]. Therefore, interventions aimed to modify these correlates may influence the way the residents perceived their neighborhood.

## Conclusions

This study has shown that the accessibility of leisure structures such as soccer fields and public squares and the accessibility of health service structures such as primary healthcare units were the environmental variables that were perceived as associated with the engagement in walk among a sample of elderly Brazilians living in a district of low socioeconomic level in the city of São Paulo. Programs promoting physical activities for elderly people in similar regions should take these variables into account.

## Competing interests

The authors declare that they have no competing interests.

## Authors' contributions

This study was conceived by EPS, RSR, and AAF. The first draft of the paper and all analyses were produced by EPS. All authors edited the paper and made critical contributions to the paper.
